# Equity, diversity, inclusion, and Indigenous training for pharmacy professionals

**DOI:** 10.1177/17151635241309016

**Published:** 2025-01-24

**Authors:** Metreyi Vishembera

**Affiliations:** Leslie Dan Faculty of Pharmacy, University of Toronto, ON

Being in community pharmacy practice for more than 7 years, I was introduced to the terms Equity, Diversity, Inclusion (EDI), Indigenous wellness, and cultural safety in pharmacy practice a couple of years ago as part of the Alberta College of Pharmacy’s (ACP) prescribed learning activities for pharmacists and pharmacy technicians. Being an international pharmacy graduate from India, I found it easy to relate to these concepts. This could be because I identified myself as part of an equity-deserving group and because I have faced racism and microaggressions during my journey in pharmacy practice, which may be due to my ethnicity, English proficiency, or accent. So I understood the relevance of providing culturally safe care and practicing with compassion—that is how I want everyone else to treat me, whether it be patients or my colleagues.

I joined the PharmD for Pharmacists Program at the University of Toronto the same year. During one of my remote Advanced Pharmacy Practice Experiences (APPE) rotations, I had the opportunity to review a series of modules on EDI and Indigenous Training and Resources (https://elearnhcp.ca/). These modules are part of the Association of Faculties of Pharmacy of Canada’s (AFPC) new learning management system for pharmacy professionals. The modules will be available on the website https://www.indigenouspharmacy.ca/resources-webi nars, which will house a link directing to the finalized modules.The main objective is to increase pharmacy professionals’ and students’ understanding and abilities to practice cultural safety, humility, and care for equity-deserving groups, including Indigenous Peoples. My first thought when I looked at the title of the modules was “That’s not something new to me.” However, the content in the modules surprised me and deeply affected my understanding of EDI, especially relating to Indigenous Peoples. I realized that my knowledge in this area was just surface level, which I attribute to the way this topic is usually taught (i.e., when we are taught that Indigenous Peoples face trauma and we should provide trauma-informed care vs what is the root cause of trauma, how it affects health outcomes, and how to provide care going forward).

These modules are comprehensive, with a strong foundational overview of health inequity and social determinants of health for various equity-deserving groups, the history and root causes of health inequity for Indigenous Peoples, and pharmacy practice integration of health equity, cultural safety, and inclusivity. The concepts are nicely elaborated and include various well-explained models like the coin model of privilege, the oppression tree metaphor, and the upstream–downstream model of health. The modules are engaging because of their design, which includes videos, podcasts, quizzes, patient case scenarios, and many resources for further reading.

Although these modules cover many diverse groups (including Black people; Two-Spirit, lesbian, gay, bisexual, transgender, queer, or questioning [2SLGBTQ+] people), I was particularly affected by the modules on Indigenous Peoples’ health inequities and their history. Learning about the history of colonization and forced assimilation was heartbreaking and at the same time enlightening.

There was a moment that made me wonder why as a pharmacy professional I need to know about the history. As soon as the learning unfolded, the topics on the *Indian Act*, residential schools, Indian hospitals, Sixties Scoop, health experimentation, and other assimilation tactics shook me and made it easier to comprehend intergenerational trauma and loss of faith in health care and, ultimately, the impact on health outcomes. I was extremely influenced by the Indigenous worldview (holistic, relational, community-based, spirituality-based, non-binary, cyclical idea of time) and related to it instantly. The upstream and downstream determinant of health model explained the areas of structures of health (worldviews, rights, legal orders, institutions, programs, and conduct) that influence social determinants of health and health disparities. The terms “Indigenization” and “decolonization” are described as part of the progress that has been made to these structures that affect Indigenous health and what role we can play in reconciliACTION plans. The content made me wonder why it took so long to understand the relevance of EDI teaching in the pharmacy setting. These modules are a step forward in that direction with a focus on the root causes of health inequity and the pharmacy practice integration of cultural safety and inclusivity.

I also wanted to explore my perspectives about this learning through an intersectional lens with respect to my several identities: as a settler from a racial/ethnic group, an international pharmacy graduate, a community pharmacist, and a PharmD student.

## Settler from racial/ethnic group

As a settler from India, I found the experience relatable due to the common colonial history and its implications. The Indigenous worldviews and the concept of holistic health resonated the most with my belief system. This learning process further strengthened my conviction to treat everyone and their belief system with respect, compassion, and humility.

## International pharmacy graduate

These modules have been more of a reflective journey as an international pharmacy graduate. My perception of Indigenous Peoples was limited to their being the original inhabitants of this land. I was ignorant about the fact that there are 3 distinct groups of Indigenous Peoples in Canada—First Nations, Métis, and Inuit—and that the Non-Insured Health Benefits (NIHB) program does not provide coverage for all Indigenous Peoples. I was not aware of Indigenous Peoples’ history, their worldviews, or health inequities. I attribute this ignorance to a lack of education about Indigenous Peoples’ colonial history and the presumption that because Canada has one of the best health care systems, it is equitable for all. This realization provides an insight into the importance of including EDI and Indigenous training as part of the assessment in licencing examinations. For example, an Objective Structured Clinical Examination (OSCE) station based on cultural safety involving a standardized patient (Indigenous) could help prepare students for real pharmacy practice.

## Community pharmacist

The experience with these modules felt like new learning from this perspective. I must acknowledge that EDI learning, as it relates to Indigenous Peoples in the pharmacy setting, seemed more meaningful. I appreciate the need to navigate NIHB coverage for my patients and address my underlying biases from my pharmacy experience (e.g., not willing to pay for medications; substance use). I feel responsible for encouraging pharmacist interns and my colleagues in my community pharmacy practice to undertake these modules as part of continued learning. The modules include the United Nations Declaration on the Rights of Indigenous Peoples (UNDRIP) articles 23 and 24 (https://www.un.org/development/desa/indigenouspeoples/wp-content/uploads/sites/19/2018/11/UNDRIP_E_web.pdf), which have motivated me to involve Indigenous Peoples in decision-making regarding their health and to respect and support their use of Traditional Medicines. The knowledge gained about the medicine wheel, 4 sacred medicines, and smudging is a small step in my journey toward increasing cultural competency. This new learning makes me feel more confident and determined to provide culturally safe and trauma-informed care. Also, ACP has emphasized the concept of person-centredness (including Indigenous patients and culture) in the recently revised standards of practice for pharmacists and pharmacy technicians, effective February 1, 2025. I am excited that I am already prepared for these new standards.

## PharmD student

As a student, I have found my experience with the modules stimulating. I now understand and value the importance of land acknowledgments (i.e., in lectures and presentations). I strongly desire to contribute to the Truth and Reconciliation Commission’s Calls to Action 23 and 24 (https://www2.gov.bc.ca/assets/gov/british-columbians-our-governments/indigenous-people/aboriginal-peoples-documents/calls_to_action_english2.pdf) I intend to continue my education and advocate for health equity and wellness for all equity-deserving groups, especially Indigenous Peoples. My first step on this journey starts with this commentary!

